# Mathematical Modelling in Biomedicine: A Primer for the Curious and the Skeptic

**DOI:** 10.3390/ijms22020547

**Published:** 2021-01-07

**Authors:** Julio Vera, Christopher Lischer, Momchil Nenov, Svetoslav Nikolov, Xin Lai, Martin Eberhardt

**Affiliations:** 1 Laboratory of Systems Tumor Immunology, Comprehensive Cancer Center Erlangen and Deutsches Zentrum Immuntherapie (DZI), Department of Dermatology, FAU Erlangen-Nürnberg, Universitätsklinikum Erlangen, 91054 Erlangen, Germany; chirstopher.lischer@uk-erlangen.de (C.L.); Xin.Lai@uk-erlangen.de (X.L.); Martin.Eberhardt@uk-erlangen.de (M.E.); 2 Institute of Mechanics, Bulgarian Academy of Sciences, Acad. G. Bonchev Str., bl. 4, 1113 Sofia, Bulgaria; m.nenov@imbm.bas.bg (M.N.); s.nikolov@imbm.bas.bg (S.N.)

**Keywords:** scientific method, computational modelling, systems biology, biomathematics, data-driven mathematical modelling, computational drug discovery, mathematical oncology

## Abstract

In most disciplines of natural sciences and engineering, mathematical and computational modelling are mainstay methods which are usefulness beyond doubt. These disciplines would not have reached today’s level of sophistication without an intensive use of mathematical and computational models together with quantitative data. This approach has not been followed in much of molecular biology and biomedicine, however, where qualitative descriptions are accepted as a satisfactory replacement for mathematical rigor and the use of computational models is seen by many as a fringe practice rather than as a powerful scientific method. This position disregards mathematical thinking as having contributed key discoveries in biology for more than a century, e.g., in the connection between genes, inheritance, and evolution or in the mechanisms of enzymatic catalysis. Here, we discuss the role of computational modelling in the arsenal of modern scientific methods in biomedicine. We list frequent misconceptions about mathematical modelling found among biomedical experimentalists and suggest some good practices that can help bridge the cognitive gap between modelers and experimental researchers in biomedicine. This manuscript was written with two readers in mind. Firstly, it is intended for mathematical modelers with a background in physics, mathematics, or engineering who want to jump into biomedicine. We provide them with ideas to motivate the use of mathematical modelling when discussing with experimental partners. Secondly, this is a text for biomedical researchers intrigued with utilizing mathematical modelling to investigate the pathophysiology of human diseases to improve their diagnostics and treatment.

## 1. Science: A World of Systems (and Models)

A good portion of science relies on the cascades of models that represent our reality at different spatial and temporal scales. A model is a simplified and often abstract representation of a complex natural system. Models are used in science to help understand, hypothesize about, or simulate the behavior of the natural systems they represent. Since a model is an abstraction of the natural system, it often includes only those elements, interactions, and processes of the system that are required to investigate the hypotheses in question. By exploiting the innate ability of the human brain to work with abstractions, models make highly complex phenomena accessible to study. In this sense, they can be used as a tool to develop hypotheses and to conceive and execute experiments to test those hypotheses. Models also provide an overview of the current knowledge on the natural system in question, facilitating the exchange of up-to-date information between researchers working on the same topic. Moreover, models can be used to simulate aspects of the natural system. A simulation is a rule-based recapitulation of the natural system’s behavior under relevant conditions using the model.

Since the features of a model are tightly linked to the purpose we want to give to it, there are multiple types of models. Models that are common to any branch of science are **semantic models**; these consist in verbalization of the natural system’s features and the hypothesis using natural language. This is the type of model that underlies the results and discussion sections in most scientific papers.

**Figure 1:**
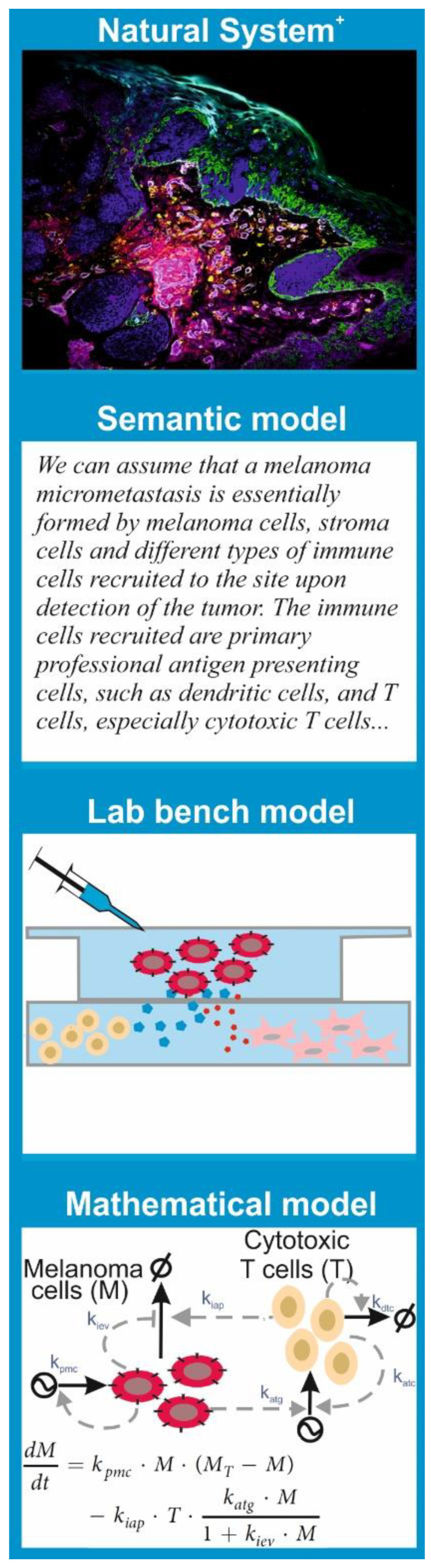
Science employs different types of models to represent natural systems. Let us suppose that we are interested in investigating the properties and potential vulnerabilities of a melanoma metastasis (here, the “**natural system**”, visualized with MELC microscopy as in Ostalecki et al. 2017 [1]). One can represent the natural system with a **semantic model**, that is, the verbalization in natural language of the key compounds and processes as well as the hypotheses about a melanoma micrometastasis. Under some simplifying assumptions, a melanoma metastasis can be studied with **lab bench models**. For example, if one is interested in the interplay between cancer and immune cells, it is possible to co-culture tumor cells with relevant types of immune cells in vitro, like in Vescovi et al. 2019 [2]. In most cases, an alternative option is **mathematical models**, that is, sets of parametric equations that encode the key properties of metastasis and the hypotheses. The mathematical model is the basis for computational simulations to design experiments or to formulate or explore hypotheses like in Santos et al. 2016 [3].

More important for our discussion are the “lab bench” models and the “mathematical” models (Figure 1). **A lab bench model** is a simplified or analogous version of a natural system employed in experiments under controlled conditions. In molecular and cell biology, lab bench models are usually units of life that can be conveniently propagated and studied in experiments to understand a given biological phenomenon. The consensus is that discoveries made via experimentation with the lab bench model provide insights into the behavior of similar phenomena in other organisms, especially humans. This is the case for cell lines, organoids, and mouse or rat strains with given genotypes or phenotypes, which have been used consistently in biomedicine as models for many human diseases.

**A mathematical model** is a set of parametric equations or other mathematical entities that encode the basic properties of the investigated natural system and that can be used to perform computational simulations. The same way that there are lab bench models with different features and purpose, there are different classes of mathematical models. One can classify them based on their treatment of the system’s dynamics as *static models*, which describe the system’s state at a point in time; *comparative static models*, which compare the properties of the system at different points in time; and *dynamic models*, which follow changes in the system over time. One can also classify models based on the mathematical *apparatus* they employ or the knowledge they exploit. There are mathematical models grounded in statistics that are used to process, analyze, and impute quantitative data generated in lab bench experiments or obtained from patient samples. All hypothesis tests and estimators of statistical correlation or inference between biological data sets are essentially (bio)statistical models.

However, there are also mathematical models that encode a mechanistic description of the natural system. This means the model equations and variables account for the interactions between the system’s key biological components and can be used for computational simulation of molecular and cellular processes. There are many subclasses of these models. A significant number of them reside in biophysics, developed to allow physically accurate simulations of atomic movement and molecular interactions in biomolecules including protein–protein interactions, DNA folding, or biomembrane dynamics. Other mechanistic models describe biochemical processes that shape cell phenotypes and cell-to-cell interactions. These are inspired by the mathematical models built in chemistry to understand and predict the kinetics of chemical reactions. In any case, just as one cannot elucidate all the mysteries of modern biomedicine with a single experimental technique, say confocal microscopy, a single subclass of mathematical model is not useful for every purpose. Every problem or hypothesis requires a carefully selected mathematical modelling approach.

## 2. The Scientific Method and the Role of Mathematical Modelling in It

Contemporary science consists in application of the scientific method and careful examination of the results one obtains with it. In the classical view, the scientific method is composed of three steps: observation of a natural phenomenon, elaboration of a hypothesis based on the observation, and design of an adequate experiment to test the hypothesis. If this is the way one would conceive scientific work nowadays, we would be strictly following the approaches employed by Galileo Galilei, Johannes Kepler, and Isaac Newton at the inception of modern science [4].

However, if you are a 21st century scientist, you are probably implementing a subtle variation of this method, which we will call the Einstein-grade scientific method. Here, the work is not performed in long-term isolation of a scientific ivory tower. Rather, the scientist continuously interacts with a community of peers. We retain the three basic steps mentioned above, but after the experimental test, one (more or less) immediately communicates the results in a booklet-length scientific publication. Early communication allows for your peers to try and reproduce your results. When experimental evidence accumulates and the underlying hypotheses are accepted, they are integrated in the scientific *corpus* of the field, that is, the set of theories, data, and hypotheses commonly accepted by the majority of researchers. Furthermore, new observations and experiments are continuously cross-checked against the scientific *corpus* in a way such that the method has an actual cyclic structure.

**Figure 2:**
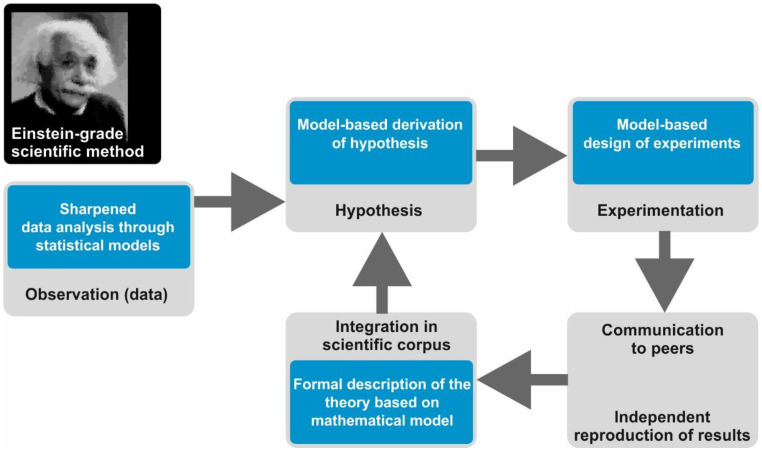
Sketch of the Einstein-grade scientific method (grey boxes) and the place that mathematical modelling occupies in it (blue boxes): the photograph of Albert Einstein is modified from the photo “Albert Einstein colorized” by Michael W. Gorth as stored in Wikimedia (CC-BY-SA-4.0, accession date: 10.08.2020; https://commons.wikimedia.org/wiki/File:Albert_Einstein_colourised_portrait.jpg).

In the Einstein-grade scientific method, mathematical and computational modelling plays a pivotal role in many different manners (Figure 2). Firstly, today’s “observations” in the majority of fields of natural sciences, including biomedicine, come as quantitative data that have to be processed, assessed, and analyzed with statistical models. In biomedicine, this is especially true in the case of sequencing data for the detection of common patterns in the sequence or the expression of genes in large cohorts of patients, a task impossible without sophisticated statistical and bioinformatics methods [5]. The features of these special mathematical models have a significant impact on the conclusions one can derive from sequencing data.

Secondly, computational models can be utilized to derive, ponder on, or substantiate hypotheses. Mathematical modelling has been employed to design validation experiments or to evaluate whether the results of the experiment agree with the expectations derived from the hypothesis and the scientific *corpus*. Finally, models can be used to achieve a formalized and unambiguous description of a field’s accepted knowledge in which theories, data, and hypotheses are organized and interconnected through equations. A remarkable example of this power is contained in a recent study by Meyer-Hermann and colleagues, in which a comprehensive mathematical model was used to summarize and elaborate around the contemporary knowledge on B-cell differentiation and maturation in germinal centers [6]. In one sense, Meyer-Hermann’s model is conceptually close to the equations that make up large physical theories, such as electromagnetism. In Supplementary Table S1, we include and comment on select publications in which the different manners to deploy models are illustrated for biomedicine.

There are a few more issues to mention and discuss about the role of mathematical models in the scientific method. The formal language of a mathematical model enhances precision and clarity compared with natural-language descriptions in the semantic models usually preferred by biomedical experimentalists. Everybody with adequate training can develop an identical understanding of a statement formulated by an equation, for example, *v* = *v_max_* * *S*/(*K_M_* + *S*). However, statements made in natural languages can be vague and can provoke misunderstandings. For example, is there a difference between “transcription factor X **activates** the expression of gene Y” and “transcription factor X **promotes** the expression of gene Y”?

The Einstein-grade method constitutes a collective endeavor. This means that it is common to find several researchers from different research institutions and locations and even with backgrounds complementing each other completing one cycle of the scientific method. For example, a German theoretical biologist, a sort of mathematical modeler, can publish a hypothesis based on his modelling efforts which will years later pique the interest of an Indonesian molecular biologist to perform experimental validation. Such occurrences are rather common in physics or chemistry, with a world record in some predictions made by Albert Einstein hundreds of years ago that have been experimentally validated only recently (which is why we use the term Einstein-grade here). However, they can also happen in biomedicine: Hodgkin and Huxley established in the first half of the 20th century a long-lasting scientific collaboration to elucidate the biophysical and biochemical mechanisms behind the initiation and propagation of action potential in nerve cell axons, a key phenomenon in understanding the ability of the nervous system to process and store information. The remarkable culmination of this collaboration was the amalgamation of their discoveries into a mathematical model which formalizes, explains, and quantifies the electrical excitability in nerve cells (see their seminal paper in Alan Hodgkin and Andrew Huxley 1952 [7] and a historical analysis in Schwiening 2012 [8]). Interestingly, some of the assumptions and simulations in the Hodgkin–Huxley model led Hodgkin’s team to predict already in 1955 that potassium ion channels can be occupied by multiple ions simultaneously. This prediction was confirmed using X-ray crystallography only in 1998, more than 40 years later, in a paradigmatic instance of the Einstein-grade scientific method in neurobiology [9].

Lastly, the scientific method is nowadays not monolithic and there are multiple variations of the general workflow commented on here [10]. This plurality of scientific methods may correspond to the different phases of discovery in diverse disciplines, the nature of the problems tackled, or even the styles of thinking belonging to different scientific communities. This notion applies also to mathematical modelling in biology and biomedicine [11].

## 3. The Love-and-Hate Relationship of Biology and Mathematics

The story of mathematics and modelling in biology is rather long, which might come as a surprise to many researchers. Biostatistical modelling and analysis have always been a key method in fields like evolutionary biology. In the beginning of the 20th century and right after the rediscovery of the Mendelian inheritance theory, there were many fundamental contributions to biology from statisticians that tried to bridge the gap between Mendelian genetics and Darwin’s theory of evolution. For example, the journal *Biometrika* [12] was established as early as 1901 by several founding fathers of modern statistics like Francis Galton and Karl Pearson with the intention of promoting biometrics, that is, the application of statistics to the analysis of biological data. Nowadays, it is difficult to dive into advanced concepts of evolutionary biology and population genetics without an understanding of mathematical modelling [13]. We also point out the seminal works of K.L. von Bertalanffy, P.A. Weiss, or M.D. Mesarovic in the application of the general systems theory to organisms dating back to the first part of 20th century (see Drack and Wolkenhauer [14] for detailed discussion).

In parallel to the search for the link between genetics and evolution theory, a new branch of science originating in physical chemistry was created by researchers interested in the dynamics of chemical reactions in living cells. This field became known as biochemistry, and already in 1913, biochemists made use of mathematical modelling to understand the mechanisms behind enzymatic catalysis. The Michaelis–Menten equation, taught in biochemistry courses throughout the world, is the first-ever mathematical model describing the dynamics of a biochemical reaction (see an updated translation of the original paper in Michaelis et al. 2011 [15]). We find in this field scientists like Jacques Monod and his team, who used mathematical modelling to understand sophisticated features of enzyme activity, such as allosteric regulation (see the seminal contribution in Monod et al. 1965 [16] and a 50-year retrospective analysis in Changeaux 2012 [17]). Fascinated by the elegance of these results, for several decades, a myriad of biochemists devoted their efforts to enzymology, that is, elucidation of the mechanisms of reaction, quantification, and modelling of the enzyme catalysis.

If mathematical modelling is necessary to immerse oneself in the connection between genes, phenotypes, and evolution as well as to understand the inner workings of catalytic proteins, how is it that certain modern biomedical researchers are reluctant to incorporate mathematical and theoretical approaches into their work? In the 1970s and 1980s, new experimental techniques were invented allowing the targeted mutation of selected genes [18]. These techniques became so fundamental in the hunt for the link between genes, proteins, and cellular functions that they led to the inception of modern molecular biology. An experimental approach based on targeted mutation followed by assessment of their effects has dominated the field since then. It is an approach that relies on advanced experimental skills, trial and error, intuition, and small-scale studies, and perhaps, that was what taught several generations of biomedical researchers that mathematics and formalized systems were somehow unnecessary in biology.

However, nowadays, two developments are turning this trend. On the one hand, newly discovered techniques for producing quantitative high-throughput data on whole classes of biomolecules (the omics revolution) required statistical methodologies for processing and analyzing these massive amounts of data. Additionally, mathematical and computational methods are indispensable in finding insights and connections between genes in these data [19]. This is the rationale behind medical genomics, the field that scans quantitative high-throughput data to find the genetic causation of diseases. On the other hand, there is mounting evidence that proteins and genes in cells do not work in isolation but rather organize into tightly interconnected networks which are often disturbed in pathological conditions [20]. These networks contain feedback loops, feedforward loops, or network hubs and gene regulatory circuits that can induce nonlinear behavior like homeostasis, self-sustained oscillations, or biostability [21]. There features, rather than anecdotic, are intrinsic and necessary to many vital cellular processes, e.g., the cell cycle [22]. 

The situation in 2020 is peculiar. We are rapidly conjuring biomedicine that necessarily relies more and more on quantitative high-throughput data, advanced statistics, bioinformatics, and computational modelling [23]. However, a significant fraction of its practitioners have insufficient mathematical and computational skills, probably ones worse than the generation that was initiated into the quantification of enzyme catalysis through kinetic equations and has recently retired. In line with this and to substantiate further discussion, we will now introduce the basics of mathematical modelling of biochemical networks.

## 4. A Primer on Mechanistic Modelling of Biochemical Systems

**Figure 3:**
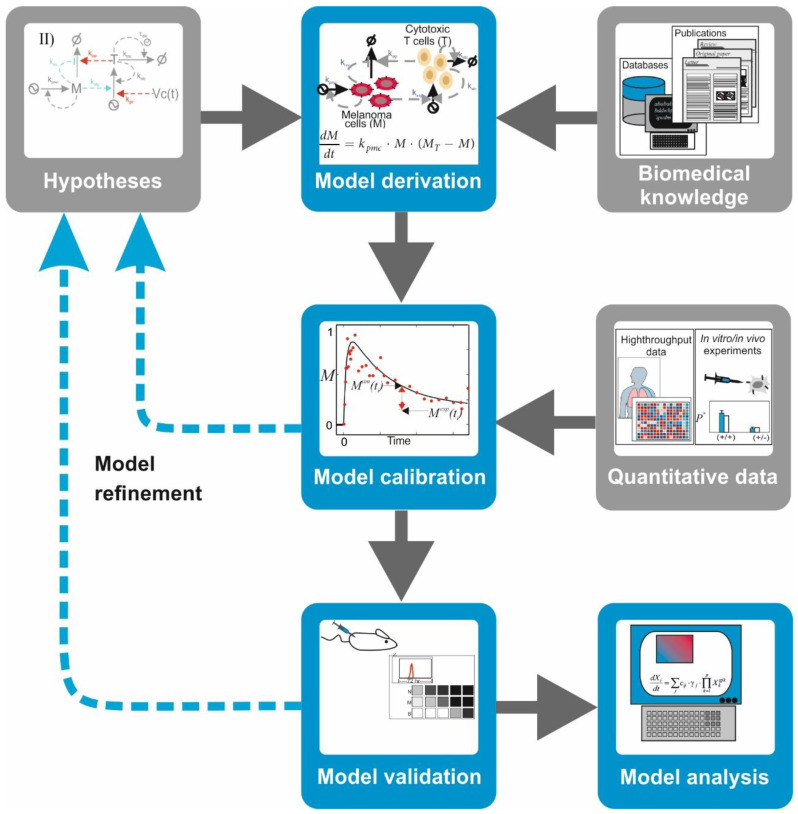
**The modelling workflow in biomedicine:** during model derivation, the biological knowledge and hypotheses about the studied system are encoded in a mathematical model. In model calibration, quantitative experimental data are added to characterize the mathematical model and to give values to the model parameters. In model validation, the ability of the model to make predictions is assessed by judging the agreement between new quantitative data and equivalent simulations of the calibrated model. In model analysis, a validated model is used to investigate the system using computer simulations or other tools like stability analysis.

There are mathematical and computational models that encode a mechanistic description of the natural system in which equations and variables account for the interactions between the key biological components of the system, for example, signaling proteins and transcription factors. Among them, there is a family of models that are conceived to integrate the topology of biochemical networks and the kinetics of their molecular interactions with their ability to control cell phenotypes. These models are inspired by the models built in chemistry to understand and predict the kinetics of chemical reactions. These mathematical and computational models are formulated, characterized, and utilized following a well-established procedure common to several branches of physics, chemistry, and engineering, which we name here as the **modelling workflow**. In a nutshell, the workflow includes the following sequential operations (Figure 3): 

**Model derivation:** Biomedical information from scientific literature is surveyed to select relevant biomolecules and interactions for the investigated hypothesis. With this information, a graphical depiction of the network of interacting molecules or cells is sketched. Under some formal or heuristic rules, a mathematical model is derived from the network graph. The mathematical model consists of mathematical equations (i.e., ordinary, partial differential, or integrodifferential equations) or other computational entities (i.e., Boolean-logic networks or Petri nets). 

**Model calibration:** To ensure that the model mimics the behavior of the natural system in a given biological scenario, one has to attribute values to free parameters in the model. In some cases, it is possible to discern these from published quantitative data. More often, however, one has to design and perform biological experiments that produce adequate quantitative data. Later, the mathematical model and the quantitative data are integrated using a computational process which assigns optimal values to the model parameters while minimizing the mismatch between experimental observations and corresponding model simulations. 

**Model validation:** The ability of the model to predict the system’s behavior is judged based on the alignment between quantitative data from a different experiment not used for calibration and the corresponding simulations of the calibrated model. A mismatch between data and simulation leads to reformulation of the hypothesis or the model’s structure, which is reflected in a modification of its mathematical equations and a re-iteration of the entire procedure. 

**Model analysis:** A validated model can be used to design and perform predictive simulations, that is, simulations of the system’s behavior under new biological scenarios. This type of simulations has been successfully deployed to detect potential drug targets or to identify biomarkers for diagnosis in cancer and other multifactorial diseases (see Table S1 for selected examples). Furthermore, tools like stability analysis and bifurcation analysis can uncover nonlinear properties of the investigated network, delineating regions in the system’s phase space with distinctive stability or critical values of the model parameters provoking qualitative changes in the system’s behavior. Despite all the power that mathematical models bring to the table, however, it goes without saying that any prediction will require further experimental validation with lab bench models. 

## 5. Frequent Unfounded Criticisms to Mathematical Modelling in Biomedicine

Now, we will list and discuss misconceptions about mathematical modelling in biomedicine which one can hear rather often when talking to experimental researchers in seminars and conferences (see also Table 1 for a summary).

### 5.1. Mathematical Models Cannot Reproduce the Complexity of Biology

When applied to biology in contrast to other natural sciences like physics or chemistry, this statement is a rather elaborate instance of magical thinking. Cells and physicochemical systems are governed by the same thermodynamic laws as any other natural system, laws that have been formulated in mathematical terms. The complexity of stellar systems’ dynamics is at least comparable with that of physiological systems, yet mathematical modelling is the standard tool to postulate hypotheses, to design experiments, and to formulate theories in astrophysics. The dynamics of the Earth’s atmosphere and climate are governed by the same laws of chemical kinetics and reaction-diffusion that apply to biochemical reactions. However, sophisticated mathematical modelling is behind the daily weather forecast or the recommendations of the Intergovernmental Panel on Climate Change. Besides nineteenth-century holistic thinking, there is not a single solid argument to support the notion that mathematical models cannot reproduce the complexity of biochemical and physiological systems.

### 5.2. Your Model Is Not Physiological. The Real System Is More Complex Than Your Mathematical Model

It may look like a softer version of the previous statement, but here, the emphasis is different. The idea is that modelling may be a valid option, but the current model does not contain sufficient detail to meaningfully represent the physiological context. When inspected more closely, this statement does not actually criticize mathematical modelling in particular but rather the fundamental act of utilizing any sort of model in biology. As we said above, any model, whether mathematical or experimental, is an abstraction that, rather than contemplating every detail of the system, includes the elements, interactions, and processes necessary to investigate the natural system under the hypothesis in question. This implies an intentional attempt at simplification by the researcher that is a shared feature of mathematical models and cell lines, organoids, or mouse models. This criticism can be countered by stating that the model must be as complex as necessary to capture the hypothesis, a notion equally valid for mathematical and lab bench models.

### 5.3. You Should Employ Data in Your Mathematical Model

A mathematical model in the sense discussed here always relies on quantitative data. As indicated above, model calibration is only possible with experimental data. Thus, a well-formulated mathematical model is based on quantitative data, which makes the above objection moot. Moreover, the interplay between model and quantitative data can come in different flavors. While usually one both calibrates and validates the model with data, one can also forego calibration in favor of analytical tools like stability analysis and can derive qualitative predictions about the system’s dynamics which are accessible to further experimental validation. Bar-or et al. 2000 [24] is a classic example of the ability of mathematical models to make qualitative predictions about the regulation of gene circuits from data. In the paper, the authors collected and synthesized all the information available at the time about the interplay between the TF p53 and its transcriptional target and repressor Mdm2 to hypothesize a) that they form a negative feedback loop gene circuit and b) that, under DNA damage, the system displayed oscillations in the expression of its components. They derived a qualitative mathematical model based on these hypotheses and found out that the model simulations predicted actual oscillations in p53 and Mdm2 levels in some experimental scenarios. They further validated this model-based prediction utilizing in vitro experiments. Interestingly, this “design principle” associated to oscillating gene circuits with TFs and their targets and repressors has been found in other master regulator TFs also by integrating quantitative data and mathematical modelling (see the case of NFkB in inflammation in Nelson et al. 2004 [25]).

### 5.4. Your Predictions Are Not Experimentally Validated

As explained above, once you perform an Einstein-grade version of the scientific method, it is not mandatory that a single paper conveys all the steps of the method. One team of researchers can formulate and investigate a hypothesis with mathematical modelling and simulations, and another team can follow up with validation experiments once technology and effort allow it (see the case of the neuronal action potential above). This is not to say that modelers are generally exempt from the need to validate, though. They should attempt to engage with experimental collaborators to see their own work come to fruition. An important aspect here is to provide ways of facilitating communication between modelers and experimentalists. In this sense, scientific papers on modelling and simulation should be written in a manner that allows the design of experiments to validate their hypotheses and predictions. Ultimately, this requires using common scientific language understandable for both mathematical modelers and experimental researchers.

### 5.5. I Do Not See the Clinical Relevance of Your Predictions

Very little of the scientific research in biomedicine delivers immediate clinical relevance, be it results obtained via mathematical modelling or through experimentation. However, at the same time, all basic research has an unpredictable long-term potential for enhancing clinical practice. To illustrate its potential, let us analyze a series of results obtained in the context of miRNA regulation and cancer. MicroRNAs exert posttranscriptional repression of selected gene targets [26] and play a pivotal role in some cell phenotypes subverted in cancer [27]. Lai et al. [28] utilized mathematical modelling to investigate the overarching hypothesis that different miRNAs can cooperate in the repression of some of their targets, a prediction that they experimentally validated for miR-572′s and miR-93′s joint repression of CDKNA1, a key cell cycle protein deregulated in cancer. In a continuation of this work, Schmitz et al. [29] used hybridization and molecular dynamics simulations of the binding of two cooperative miRNAs on their target mRNA to illustrate the general biophysical feasibility of this mechanism and to elucidate how it works at the molecular level. Moreover, they performed a human genome-wide exploration to systematically look for this type of joint miRNA regulation. Based on these results and further computational simulations, they hypothesized that miRNA cooperativity and its modelling can predict drug targets in cancer. They tested the hypothesis in a case study on cooperativity between miR-205-5p and miR-342-3p and its capacity to repress E2F1-mediated chemoresistance in cancer [30]. Their model-based prediction was confirmed experimentally. Finally, Lai et al. [31] extended this approach to the whole genome and systematically identified pairs of miRNAs that cooperatively target upregulated genes in metastatic melanoma. In summary, this series of interconnected papers illustrates how mathematical modelling can lead the way from hypothesis formulation and basic research to identifying potential clinical applications.

## 6. Rules to Build Mathematical Models That Can Be Understood by Experimentalists

To conclude, we elaborate on a few recommendations for biomedical modelers for when they conceive and implement their mathematical models, which hopefully will help bridge the cognitive chasm between them and experimentalists.

### 6.1. Know Your Problem

A good modeler should become an expert in the biomedical system that they plan to model. This is the best warranty that the structure and the hypotheses behind the mathematical model make sense and are consistent with the current biomedical knowledge. In addition, becoming an expert on the topic will help in choosing the right model assumptions, data, and hypotheses to be tested. Complementary to this, the best models emerge from constant interaction between competent biomodelers and experimental researchers. Remember, though, that collaboration is productive when communication is fluent, and this is only possible when a common language is spoken. In the present, this common language is the one that modelers need to learn when diving into the biology of the system they want to model. We refer again to Meyer-Hermann et al. 2012 [6] and their ability as modelers to acquire a deep understanding of B-cell biology and how they translated it into their mathematical model. However, we postulate that, in the long-term, biomedical researchers need in turn to rediscover the more precise language that math offers, which will also help them to quickly grasp advances in their own field of interest. There is an in-between methodology that could rekindle the growth of mathematical skills in experimentalists: network biology. In silico reconstruction, visualization, and modelling of intracellular biochemical networks provide a framework for connecting genes and molecules quantitatively to phenotypes and hence understanding the function and dynamics of cellular systems [32]. The network biology approach relies on mathematical concepts from graph theory, statistics, and mathematical modelling but is yet intuitive enough to allow a fluent discussion between wet- and dry-lab biologists. This has been, for example, advantageously used by yeast biologists to connect their experimentally detected biological interactions with their effect in cell phenotypes.

### 6.2. Select the Right Type of Mathematical Model, and Select It Early

The features of the mathematical model largely depend on the aim of the study; the scale and structural complexity of the investigated system; and the quantity, quality, and nature of the available experimental data. A model to investigate the nonlinearity associated with a feedback loop circuit has completely different requirements than a model of quantitative drug dosage in humans. This affects in particular the selection of the mathematical framework in which the model is derived and simulated. There is no single best modelling framework for every biomedical system or purpose, and therefore, the choice of model often relies on a trade-off between several requirements. We want to mention here (a) the computational demand and scalability, (b) the nature and necessary amount of calibration data, and (c) the way time and space are handled in the simulations. Sometimes, standard modelling frameworks are not suited for the problem in question and hybrid computational models of different types need to be considered (Chiam et al. 2006 illustrate how this type of hybridization can be done in the context of signaling pathways [33]; a discussion of this issue in the context of bacterial infection can be found in Cantone et al. 2017 [34]). When trying to facilitate the communication between modelers and experimentalists, one interesting approach is rule-based modelling. Compared to more math-heavy methods, rule-based modelling allows compact representations of reaction networks with a language-oriented structure; this makes them similar to semantic models and hence closer to the way of thinking of experimentalists (as a case study, see the epidermal growth factor receptor signaling network built in [35]).

### 6.3. Build on Preceding Efforts

To start the derivation of a mathematical model totally from scratch makes sense only when there is no alternative. A prudent modeler should reuse, extend, and adapt preexisting models when possible. In some cases, this will not be possible because the available models are based on different experimental conditions, formulated for a different biological scenario, or derived using an unsuitable modelling framework. In these cases, even if the model is found to be partially incompatible, its assessment will help judge the validity and portability of its assumptions and hypotheses in the context of one’s own modelling effort. If the problem lies in the modelling framework, it is sometimes worth translating the model into one’s chosen framework. In the ideal case, the model authors should have uploaded a fully annotated version of their model to a public repository (e.g., Biomodels [36]), which facilitates the work of incorporating the model into one’s own. Some tools even allow the semiautomatic translation of models from one formalism to another (see for example OdiFy [37]). As an example of this idea of building on preceding modelling efforts, in Csikász-Nagy 2009, one can find a comprehensive overview of the cascade of increasingly detailed mathematical models constructed since 1991 to understand the regulation of the cell cycle and how mathematical models are based on or have benefitted from the results obtained with previously developed models [38].

### 6.4. The Size Does Not Always Matter

**Figure 4:**
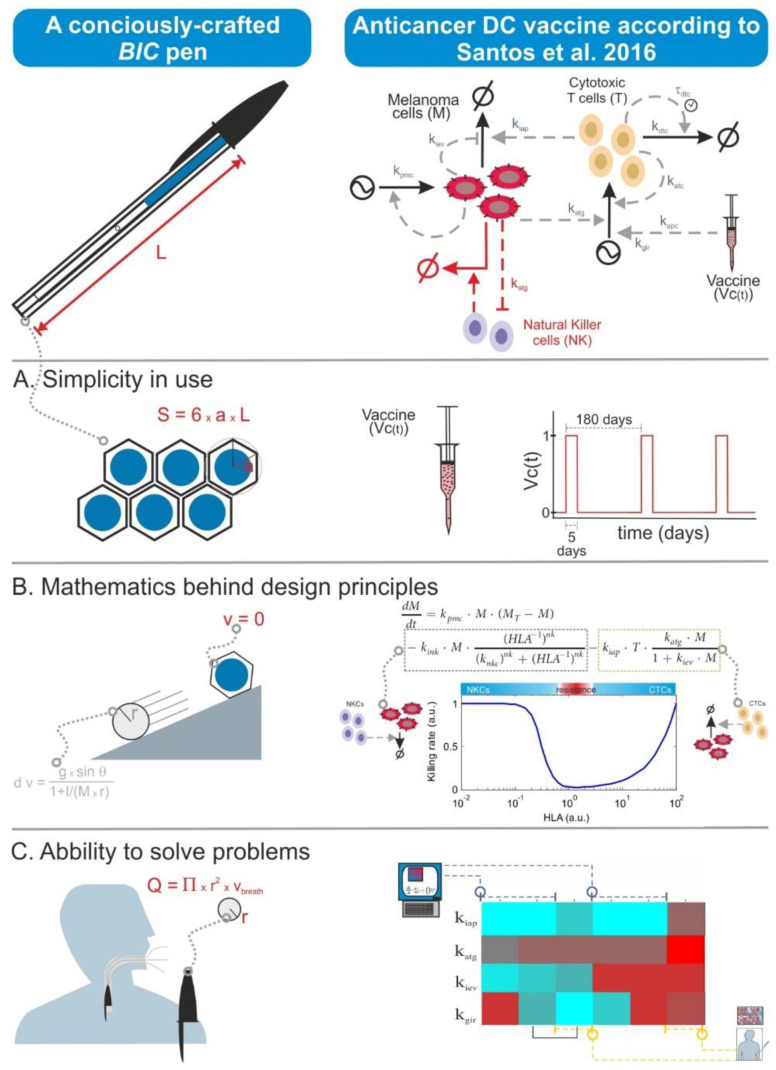
**BIC pen-like mathematical models: prioritizing the purpose of the model instead of its detailedness**. BIC pens look like the simplest and cheapest ballpoint pen one can buy, but their apparent simplicity conceals features conceived to optimize them in economic, ergonomic, and safety terms. In Santos and coworkers 2016 [3], a similar strategy was followed to build a mathematical model accounting for anticancer dendritic cell (DC) vaccination composed of only two ordinary differential equations, far simpler than other published models [41]. (**A**) **Simplicity in design:** BIC pens have a characteristically simple hexagonal structure; this apparently naïve choice significantly reduces the material consumption of the pen and minimizes the required space for storage. An important aspect to consider in DC vaccine modelling is the bioavailability of the cells after their injection. There are much elaborated models describing this process [42], but for our purpose, it was sufficient to model DC bioavailability with a cyclic piecewise linear function that mimics the known overall behavior of injected DCs. (**B**) Mathematics behind design principles: compared to standard circular pens, BIC pens hardly roll on the surface of a table. This feature was explicitly desired when drafting their design. In Santos et al., we wanted a simple enough model that was still able to mimic the interaction between the tumor and both innate and adaptive immunity; to this end, the model contained two nonlinear kinetic rates in a single equation, which are still able to mimic the basics of the interplay between the tumor and the two branches of immunity. (**C**) Ability to solve problems: in the end, simplicity has to be reconciled with effectiveness. The design of a BIC pen, for example, integrates more characteristics like minimizing the risk of suffocation when swallowing the cap. The predictions made in Santos et al. (2016) in terms of which phenotypic features sensitize the tumor to the therapy were aligned with patient data from clinical trials; furthermore, the model predicted alternative phenotypes that promote therapy resistance. The figures about DC vaccine modelling are adapted from Santos et al. 2016 under the conditions of an open access publication (CC BY 4.0). The figures about the BIC pen were inspired by the content of the webpage www.bicworld.com. Fight by doing: A route map to good mathematical modelling in biomedicine.

There is nowadays a tendency to moon-shoot everything in biomedicine. This has translated also to biomodelling, and some researchers think that the quality of a mathematical model is measured in terms of the number of model variables as well as the required computational power and the complexity of the simulation algorithms. However, quality in modelling is primarily achieved through the biological precision of the assumptions encoded in the model equations. Thus, models can look simple and be small in terms of their number of equations but can actually possess the right features for the purpose of the specific modelling effort. This is somewhat similar to a BIC ballpoint pen. This is the simplest and cheapest ballpoint pen one can buy, but it actually displays a number of easy-to-overlook sophisticated features conceived to optimize its design in economic, ergonomic, and safety terms. One can also formulate BIC-like mathematical models, in which one gives priority to the description of the biological context and hypothesis and its planned utilization rather than to unnecessary size, complexity, or levels of detail (Figure 4).

### 6.5. Set Your Results in Stone

One should prepare one’s own models in such a way that they can be understood and reused by other researchers, thereby closing the loop inherent to the scientific method. This is a frequently disregarded aspect in many fields in mathematical biomedicine, in which the number of modeler teams can be counted on two hands, and consequently, any model should have a high potential for reuse, adaptation, or integration. A model that is difficult to reproduce or understand by other modelers is guaranteed to collect virtual dust on its publication shelf. Thus, it is advantageous to implement equations and simulations in standard formats [39]. Further, whenever possible, the models and their supporting experimental data should be uploaded to repositories [36]. In closing, we emphasize the importance of the careful manual curation and annotation of mathematical models. In line with this, whenever possible, one should give preference to frameworks that allow for fast, simple, and standardized dissemination and model exchange [40].

## 7. The Best of Both Worlds—A Final Note on Mathematics, Models, Big Data, and Experimental Biology

The approach based on semantic and lab bench models has been very successful in past decades in describing many fundamental regulatory pathways in molecular biology. However, due to the massive amount of data produced nowadays, we are quickly reaching its limits. This is especially true (and dramatic) in the description of dynamic systems like heterogeneous cell populations, genomic regulation, or multifactorial diseases like cancer, for which this approach is clearly inadequate. There is no semantic solution to properly characterize multi-variable states, events, or diseases.

There is a clear understanding in the wet-lab biology community that advanced high throughput data analysis approaches are an urgent necessity for them, but in the opinion of many wet-lab biologists, data analysis and modelling are often conceived as a mere press of the button on computers that leads to publication-ready figures and plots within minutes. This has led to an entire industry producing “clickable” data analysis and modelling tools that make little demands on the knowledge of the operator. We think that this can lead to many problems, like reproducibility issues or the inability to critically judge the results produced by those tools, especially since most of them use proprietary algorithms. Additionally, the ability to generate an output from a purchased software or analysis suite might not automatically mean that this output is correct or sensible. Hence, an understanding of the underlying data and modelling theory is important and is often neglected in some wet-lab settings.

In the future, we as a community of biomedical researchers should strive to recognize that we need both sides of the coin, giving them equal weight in considerations like funding or time investment. Biology should orient itself towards the other two major natural science disciplines, physics and chemistry, and try to give their students a well-rounded education in mathematical understanding, data analysis, and modelling as well as in computer programming. Until that time, we need to make sure that we find ways to communicate between the two areas in a way that furthers productive collaboration.

## Figures and Tables

**Table 1:** Top misunderstandings on modelling and how to fight them.

**Mathematical models cannot reproduce the complexity of biology**. Cells and biochemical systems are governed by the same laws as any other physicochemical system, most of which are successfully simulated with mathematical models (see, for example, computer model-based weather and climate prediction).
**Your model is not physiological. The real system is more complex than your mathematical model**. A model must include the elements, interactions, and processes necessary to investigate the hypothesis in question (nothing else), and this is equally valid for mathematical and lab bench models.
**You should employ data in your mathematical model**. A well-formulated mathematical model is based on quantitative data taken from databases and published reports or produced for calibration of the model. Also, one can use qualitative model analysis to formulate hypotheses and propose experiments; here, the experimental data comes after the model.
**Your predictions are not experimentally validated**. Model derivation and simulation can be temporally detached from experimentation: a researcher can investigate a hypothesis with mathematical modelling and can leave the experimental validation for another team after publication of the model-based predictions.
**I do not see the clinical relevance of your predictions**. There are mathematical models that are of mandatory use in biomedicine (see pharmocokinetics models for drug approval). However, mathematical modelling is in most cases theoretical research. Similar to any other basic research approach, it has an unpredictable long-term potential for enhancing clinical practice.

## Supplementary Materials

The following are available online at https://www.mdpi.com/1422-0067/22/2/547/s1, Table S1: Real cases of mathematical modeling in biomedicine with additional bibliography and further reading.
