# Supplementary material for: Mathematical Modelling in Biomedicine: A Primer for the Curious and the Skeptic
Source: Int J Mol Sci. 2021 Jan 7;22(2):547. doi: 10.3390/ijms22020547 (PMC7826848; doi:10.3390/ijms22020547)
Supplement: Supplementary file 1 [file ijms-22-00547-s001.pdf]

Supplementary Material

Further reading

1. **Modelling to understand complex gene circuits.** One of the main motivations to go for modelling in gene circuits is the existence of feedback and feedforward loops that give rise to non-linear, counter-intuitive regulatory patterns which can scale to the tissue level [1]. Tyson and coworkers [2] is a mathematical modelling-based discussion of the sometimes unexpected properties found in frequent gene and signaling regulatory circuits. The same authors updated and extended this analysis in a more recent paper [3] and in a cancer relevant case study [4]. The systematic use of mathematical modelling to formalize the dynamics of intracellular circuits is not new. One can, for example, find a continuously revised book deploying a mathematical understanding on the topic published as early as 1976 [5].

2. **Cell cycle and oscillations.** The cell cycle is the best-known and -studied example of a molecular oscillatory circuit, and the Ferrell team has for years combined mathematical modelling and experimentation to dissect its regulation [6]. Ferrell and coworkers 2011 [7] is a perfect introduction to the way of thinking of modelers using the cell cycle as a case study to illustrate basic notions of non-linear gene circuits. Oscillations are not only a feature of the cell cycle; modeling has been intensively used to analyze oscillations in the inflammation-related signalling pathway NF-κB (see a recent example in [8]).

3. **Negative feedback loops and ERK signaling.** The Ras-Raf-MEK-ERK cascade, a pathway linked to cancer progression and aggressiveness, is a system enriched in feedback loop circuits [9]. In their 2010 review [10], Kholodenko, Kolch and colleagues beautifully illustrated the connections between the molecular features of ERK signaling and their capacity to induce sophisticated cell regulation, all of it using the modeler's mindset. Interestingly, feedback loops can generate sophisticated regulatory patterns even when operating at the single-molecule level [11].

4. **Network Biology as the bridge between pure experimentation and pure theory.** Under the network biology paradigm, *in silico* determination and modeling of intracellular regulatory networks is utilized to establish a quantitative framework for understanding the functional, logical, and dynamic aspects of cellular systems, but also the connection between gene ensembles and phenotypes [12]. Regarding human diseases, there are noticeable examples in the literature, in which this approach is used to select drug combinations for treatment of diseases [13], study relationships between genes,

interactome networks, and human diseases on a systemic level [14], or predict association of genes or genetic variants with human diseases [15,16].

5. Teaching modelling in biomedicine. If planning to introduce mathematical modelling in undergraduate lectures, the book by E.O. Voit is an excellent introductory material based on Voit's long experience in teaching systems biology to engineers and biomedical researchers [17].

Table S1. Real cases of mathematical modeling in biomedicine

Mathematical models to process, assess, and analyze HTD data. Advanced statistical models are behind the analysis of genomics data from large patient cohorts. The Pan-Cancer Analysis of Whole Genomes (PCAWG) Consortium recently deployed a combination of biostatistics, bioinformatics, and computational biology models to integrate information taken from 2,658 whole-cancer genomes. It barely merits mention that without intensive use of mathematical tools this analysis would have been impossible [18].
Derivation or substantiation of hypotheses. The Gatenby team has focused for nearly three decades on mathematical modelling to understand cancer progression and therapy [19]. In a recent paper, they took advantage of computational modelling and game theory to substantiate the hypothesis that cancer progression can be delayed in resistant prostate cancer treated with inhibitors of testosterone auto-production enzymes if one considers the interplay between androgen-dependent, androgen-producing, and androgen-independent tumor cells [20]. Further experimental data confirmed their hypothesis.
Design of experiments aiming at validating hypothesis. Model simulation and analysis can be employed to point to variables (molecules or processes) that play a critical role in understanding the dynamics of the investigated cellular system, in particular when the variables are difficult to track experimentally and would otherwise be neglected. In Vera and colleagues 2013 [21], mathematical modelling describing the dynamics of tumor cells under deregulation of a gene circuit composed of TFs and miRNAs led to the design of *in vitro* experiments. The model simulations suggested that conventional anticancer drug treatment would select cancer cells displaying a resistant genotype based on deregulation of a given TF-miRNA circuit, a prediction that was confirmed in the *in vitro* experiments. Becker *et al.* developed a data-driven model to investigate hypothetical mechanisms that account for dynamic processes of the erythropoietin receptor (EpoR) activation, such as receptor mobilization, recycling, and turnover. With the help of model simulations and analysis, the authors experimentally validated that rapid erythropoietin depletion and compensation of endocytic removal of cell surface EpoR by receptor turnover is an adaptable mechanism for enabling cells to respond to variation of erythropoietin concentrations over an extreme range [22].
Formal and structured description of the knowledge of a scientific field. The Meyer-Hermann team has been modelling B cell function for a decade [23]. In Meyer-Hermann and coworkers 2012 [24], the authors developed a comprehensive mathematical model of B cell selection, division, and exit from the germinal center. This mathematical model is utilized to summarize current knowledge as well as to discuss new hypotheses about B cell differentiation. The authors further employed the model to interpret key events in B cell biology and to formulate predictions about them.
Drug discovery. The use of computational models is essential in pharmacology to quantify drug bioavailability and distribution, and to establish secure drug dosage. But mathematical modelling can be used beyond that to predict drug targets and design or refine drugs. In Schoeberl and colleagues 2009 [25], a team from a biotechnological company searched for drug targets for the ErbB-PI3K signaling pathway, which is commonly subverted in solid tumors, with quantitative computational modelling and sensitivity analysis. In a second paper eight years later,

they discussed the entire process that brought them from computer modelling to the clinical trial of an actual anti-ErbB3 antibody.

Biomarker discovery. Khan and coworkers reconstructed a large network illustrating the role of the E2F transcription factor family in the triggering of cancer phenotypes [26]. When they combined RNA-Seq from aggressive cell lines and computer algorithms with the network, they isolated a core gene circuit linked to aggressiveness. Mathematical modelling and simulation of this core circuit derived a gene signature linked to cancer aggressiveness. The signature was subsequently validated with *in vitro* experiments and in comparisons with patient datasets.

Simulation-based diagnostics. The multidisciplinary team led by Markus Morrison and Jochen Prehn has explored for more than a decade quantitative profiling of tumor biopsies and mathematical modelling in cancer diagnostics. Their focus is on predicting the response to chemotherapy in solid tumors by assessing the responsiveness of the apoptosis pathway to the therapy. In their incremental efforts, they first developed biologically insightful mathematical models [27,28], and then adapted them to a clinical setup with remarkable success [29,30].

Mathematical models to think beyond current knowledge and hypotheses. Mathematical modelling can be employed in biomedicine in the same way as in physics, to motivate or substantiate disruptive hypotheses. Deploying a modeler's perspective, Nikolov and coworkers hypothesized that tumors resemble complex dynamical systems known as chaotic attractors and elaborated the consequences of this assumption for our understanding of cancer progression [31,32]. Further, models can help connect biological experiments and data from different temporal and organizational scales (for a model connecting intracellular and cell-to-cell networks, see for example [33]).

Bibliography

1. Raspopovic, J.; Marcon, L.; Russo, L.; Sharpe, J. Modeling Digits. Digit Patterning Is Controlled by a Bmp-Sox9-Wnt Turing Network Modulated by Morphogen Gradients. *Science* **2014**, *345*, 566–570, doi:10.1126/science.1252960.
2. Tyson, J.J.; Chen, K.C.; Novak, B. Sniffers, Buzzers, Toggles and Blinkers: Dynamics of Regulatory and Signaling Pathways in the Cell. *Curr. Opin. Cell Biol.* **2003**, *15*, 221–231, doi:10.1016/S0955-0674(03)00017-6.
3. Tyson, J.J.; Novák, B. Functional Motifs in Biochemical Reaction Networks. *Annu. Rev. Phys. Chem.* **2010**, *61*, 219–240, doi:10.1146/annurev.physchem.012809.103457.
4. Tyson, J.J.; Baumann, W.T.; Chen, C.; Verdugo, A.; Tavassoly, I.; Wang, Y.; Weiner, L.M.; Clarke, R. Dynamic Modelling of Oestrogen Signalling and Cell Fate in Breast Cancer Cells. *Nat. Rev. Cancer* **2011**, *11*, 523–532, doi:10.1038/nrc3081.
5. Savageau, M.A.; Rosen, R. *Biochemical Systems Analysis: A Study of Function and Design in Molecular Biology*; Addison-Wesley Pub. Co, Advanced Book Program: Reading, MA, USA, 1976;
6. Chang, J.B.; Ferrell, J.E. Mitotic Trigger Waves and the Spatial Coordination of the Xenopus Cell Cycle. *Nature* **2013**, *500*, 603–607, doi:10.1038/nature12321.
7. Ferrell, J.E.; Tsai, T.Y.-C.; Yang, Q. Modeling the Cell Cycle: Why Do Certain Circuits Oscillate? *Cell* **2011**, *144*, 874–885, doi:10.1016/j.cell.2011.03.006.
8. Zambrano, S.; De Toma, I.; Piffer, A.; Bianchi, M.E.; Agresti, A. NF-KB Oscillations Translate into Functionally Related Patterns of Gene Expression. *eLife* **2016**, *5*, e09100, doi:10.7554/eLife.09100.
9. Sturm, O.E.; Orton, R.; Grindlay, J.; Birtwistle, M.; Vyshemirsky, V.; Gilbert, D.; Calder, M.; Pitt, A.; Kholodenko, B.; Kolch, W. The Mammalian MAPK/ERK Pathway Exhibits Properties of a Negative Feedback Amplifier. *Sci. Signal.* **2010**, *3*, ra90, doi:10.1126/scisignal.2001212.
10. Kholodenko, B.N.; Hancock, J.F.; Kolch, W. Signalling Ballet in Space and Time. *Nat. Rev. Mol. Cell Biol.* **2010**, *11*, 414–426, doi:10.1038/nrm2901.
11. Hernansaiz-Ballesteros, R.D.; Cardelli, L.; Csikász-Nagy, A. Single Molecules Can Operate as Primitive Biological Sensors, Switches and Oscillators. *BMC Syst. Biol.* **2018**, *12*, 70, doi:10.1186/s12918-018-0596-4.
12. Vidal, M.; Cusick, M.E.; Barabási, A.-L. Interactome Networks and Human Disease. *Cell* **2011**, *144*, 986–998, doi:10.1016/j.cell.2011.02.016.
13. Cheng, F.; Kovács, I.A.; Barabási, A.-L. Network-Based Prediction of Drug Combinations. *Nat. Commun.* **2019**, *10*, 1197, doi:10.1038/s41467-019-09186-x.
14. Goh, K.-I.; Cusick, M.E.; Valle, D.; Childs, B.; Vidal, M.; Barabási, A.-L. The Human Disease Network. *Proc. Natl. Acad. Sci. U. S. A.* **2007**, *104*, 8685–8690, doi:10.1073/pnas.0701361104.
15. Maiorino, E.; Baek, S.H.; Guo, F.; Zhou, X.; Kothari, P.H.; Silverman, E.K.; Barabási, A.-L.; Weiss, S.T.; Raby, B.A.; Sharma, A. Discovering the Genes Mediating the Interactions between Chronic Respiratory Diseases in the Human Interactome. *Nat. Commun.* **2020**, *11*, 811, doi:10.1038/s41467-020-14600-w.
16. Yadav, A.; Vidal, M.; Luck, K. Precision Medicine - Networks to the Rescue. *Curr. Opin. Biotechnol.* **2020**, *63*, 177–189, doi:10.1016/j.copbio.2020.02.005.
17. Voit, E. *A First Course in Systems Biology*; 2017; ISBN 978-0-8153-4568-8.
18. ICGC/TCGA Pan-Cancer Analysis of Whole Genomes Consortium Pan-Cancer Analysis of Whole Genomes. *Nature* **2020**, *578*, 82–93, doi:10.1038/s41586-020-1969-6.
19. Gatenby, R.A. Population Ecology Issues in Tumor Growth. *Cancer Res.* **1991**, *51*, 2542–2547.

20. Zhang, J.; Cunningham, J.J.; Brown, J.S.; Gatenby, R.A. Integrating Evolutionary Dynamics into Treatment of Metastatic Castrate-Resistant Prostate Cancer. *Nat. Commun.* **2017**, *8*, 1816, doi:10.1038/s41467-017-01968-5.
21. Vera, J.; Schmitz, U.; Lai, X.; Engelmann, D.; Khan, F.M.; Wolkenhauer, O.; Pützer, B.M. Kinetic Modeling-Based Detection of Genetic Signatures That Provide Chemoresistance via the E2F1-P73/DNp73-MiR-205 Network. *Cancer Res.* **2013**, *73*, 3511–3524, doi:10.1158/0008-5472.CAN-12-4095.
22. Becker, V.; Schilling, M.; Bachmann, J.; Baumann, U.; Raue, A.; Maiwald, T.; Timmer, J.; Klingmüller, U. Covering a Broad Dynamic Range: Information Processing at the Erythropoietin Receptor. *Science* **2010**, *328*, 1404–1408, doi:10.1126/science.1184913.
23. Meyer-Hermann, M.; Deutsch, A.; Or-Guil, M. Recycling Probability and Dynamical Properties of Germinal Center Reactions. *J. Theor. Biol.* **2001**, *210*, 265–285, doi:10.1006/jtbi.2001.2297.
24. Meyer-Hermann, M.; Mohr, E.; Pelletier, N.; Zhang, Y.; Victora, G.D.; Toellner, K.-M. A Theory of Germinal Center B Cell Selection, Division, and Exit. *Cell Rep.* **2012**, *2*, 162–174, doi:10.1016/j.celrep.2012.05.010.
25. Schoeberl, B.; Pace, E.A.; Fitzgerald, J.B.; Harms, B.D.; Xu, L.; Nie, L.; Linggi, B.; Kalra, A.; Paragas, V.; Bukhalid, R.; et al. Therapeutically Targeting ErbB3: A Key Node in Ligand-Induced Activation of the ErbB Receptor-PI3K Axis. *Sci. Signal.* **2009**, *2*, ra31, doi:10.1126/scisignal.2000352.
26. Khan, F.M.; Marquardt, S.; Gupta, S.K.; Knoll, S.; Schmitz, U.; Spitschak, A.; Engelmann, D.; Vera, J.; Wolkenhauer, O.; Pützer, B.M. Unraveling a Tumor Type-Specific Regulatory Core Underlying E2F1-Mediated Epithelial-Mesenchymal Transition to Predict Receptor Protein Signatures. *Nat. Commun.* **2017**, *8*, 198, doi:10.1038/s41467-017-00268-2.
27. Rehm, M.; Huber, H.J.; Dussmann, H.; Prehn, J.H.M. Systems Analysis of Effector Caspase Activation and Its Control by X-Linked Inhibitor of Apoptosis Protein. *EMBO J.* **2006**, *25*, 4338–4349, doi:10.1038/sj.emboj.7601295.
28. Schmid, J.; Dussmann, H.; Boukes, G.J.; Flanagan, L.; Lindner, A.U.; O'Connor, C.L.; Rehm, M.; Prehn, J.H.M.; Huber, H.J. Systems Analysis of Cancer Cell Heterogeneity in Caspase-Dependent Apoptosis Subsequent to Mitochondrial Outer Membrane Permeabilization. *J. Biol. Chem.* **2012**, *287*, 41546–41559, doi:10.1074/jbc.M112.411827.
29. Hector, S.; Rehm, M.; Schmid, J.; Kehoe, J.; McCawley, N.; Dicker, P.; Murray, F.; McNamara, D.; Kay, E.W.; Concannon, C.G.; et al. Clinical Application of a Systems Model of Apoptosis Execution for the Prediction of Colorectal Cancer Therapy Responses and Personalisation of Therapy. *Gut* **2012**, *61*, 725–733, doi:10.1136/gutjnl-2011-300433.
30. Salvucci, M.; Würstle, M.L.; Morgan, C.; Curry, S.; Cremona, M.; Lindner, A.U.; Bacon, O.; Resler, A.J.; Murphy, Á.C.; O'Byrne, R.; et al. A Stepwise Integrated Approach to Personalized Risk Predictions in Stage III Colorectal Cancer. *Clin. Cancer Res. Off. J. Am. Assoc. Cancer Res.* **2017**, *23*, 1200–1212, doi:10.1158/1078-0432.CCR-16-1084.
31. Nikolov, S.; Wolkenhauer, O.; Vera, J. Tumors as Chaotic Attractors. *Mol. Biosyst.* **2014**, *10*, 172–179, doi:10.1039/c3mb70331b.
32. Nikolov, S.; Dimitrov, A.; Vera, J. Hierarchical Levels of Biological Systems and Their Integration as a Principal Cause for Tumour Occurrence. *Nonlinear Dyn. Psychol. Life Sci.* **2019**, *23*, 315–329.
33. Santos, G.; Vera, J. The Role of Age, Neutrophil Infiltration and Antibiotics Timing in the Severity of Streptococcus Pneumoniae Pneumonia. Insights from a Multi-Level Mathematical Model Approach. *Int. J. Mol. Sci.* **2020**, *21*, 8428, doi:10.3390/ijms21228428.
